# Microwave and Steam Processing: A Novel Approach to Modifying the Characteristics of Reconstituted Whole Wheat Flour and Dough

**DOI:** 10.3390/molecules30020203

**Published:** 2025-01-07

**Authors:** Xuhua Liu, Qiangqiang Sun, Rong Yan, Yaqing Wang, Junying Wang, Liping Yang, Ligong Zhai

**Affiliations:** 1 Food Science and Engineering College, Anhui Science and Technology University, Chuzhou 233100, China; liuxuhua225@163.com (X.L.); 19556908376@163.com (Q.S.); yanrongm556@163.com (R.Y.); wyq36990121@163.com (Y.W.); wangjy@ahstu.edu.cn (J.W.); yanglp@ahstu.edu.cn (L.Y.); 2 Forestry College, Nanjing Forestry University, Nanjing 210037, China

**Keywords:** whole wheat flour, wheat bran, modification treatment, processing properties

## Abstract

To reduce the adverse effects of bran on whole wheat flour products. In this study, seven reconstituted whole wheat flours were prepared and used to determine the effects of microwave and steam treatment on bran. We aimed to understand the effect of modification treatment on the properties of reconstituted whole wheat flour and dough. Treatment with whole wheat flour had a significant impact on the color, solubility, and swelling. As the cooking time increased, the initial temperature (To), peak temperature (Tp), and final temperature (Tc) of pasting and enthalpy (Hp) decreased. The combination of microwave and steam modification increased water absorption and stabilization time, leading to improved fermentation performance and cooking stability of the dough. The modified whole wheat flour and dough exhibited a significant decrease in crystallinity, possibly due to the degradation of the crystalline and amorphous regions of the starch granules during heat treatment. Upon modification treatment, the spiral β-turn structure was transformed into an irregular curled and β-sheet structure, and the β-sheet ratio increased significantly (*p* < 0.05). The modification of bran through microwave treatment (700 W for 30 s) followed by steam treatment (10 min) enhanced the processing performance of reconstituted whole wheat flour, offering substantial potential for the development of novel products and the optimization of industrial production efficiency.

## 1. Introduction

The long-term consumption of refined foods is closely associated with an increased risk of chronic diseases. Compared to refined grains, whole grains are rich in cellulose, hemicellulose, proteins, B vitamins, vitamin E, minerals, thiamine, riboflavin, and niacin. The fats found in whole grains mainly consist of essential saturated fatty acids, one-third of which is linoleic acid [1]. In addition, whole grains contain small amounts of plant sterols and phospholipids. The consumption of whole grains is associated with anti-obesity effects. Whole-grain consumption reduces the incidence of type II diabetes, improves insulin sensitivity, inhibits the development of cardiovascular disease, and lowers the risk of colorectal cancer [2].

Although foods made from whole wheat flour offered health benefits, consumer acceptance and consumption of whole grain products were relatively low compared to those of wheat-based products. Globally, wheat is a staple food for 2.9 billion people. There are two main reasons for this finding. First, the inclusion of wheat bran in whole wheat foods can weaken gluten, leading to a decrease in specific volume and an increase in hardness. This can result in darker colors, rougher taste, harder texture, smaller size, and unpleasant odor [3]. The outer layer of wheat particles, known as wheat bran, contains pollutants such as pesticide residues, microorganisms, mycotoxins, and heavy metals [4]. Compared to wheat flour, whole wheat flour has higher levels of fats and enzymes (such as lipase and lipoxysynthase) [5]. This reduces the shelf stability of whole-grain products, leading to reduced product quality. Therefore, it is important to develop methods to stabilize wheat bran.

Commonly used bran modification treatment technologies are categorized into physical and biological modifications. Physical modifications include techniques such as high-pressure, microwave, baking, atmospheric pressure steam, extrusion puffing, turbo process heat, and superheated steam treatments [6]. Biological modifications involve microbial fermentation using yeast or lactic acid bacteria, as well as enzymatic treatments using enzymes such as xylanase, glycoamylase, and fibrillar (vimentinase). Proper microwave modification of bran (700 W, 20 s) results in the production of whole wheat buns, which results in a good-quality product with a softer texture. Additionally, microwave treatment can deactivate lipase and lipoxygenase, thereby extending product shelf life [7]. Atmospheric-pressure steam treatment decreases the protein content of whole wheat flour. Additionally, atmospheric-pressure steam treatment increases the landing values as well as improves the peak viscosity, decay value, final viscosity, regeneration value, peak time, and pasting temperature. Furthermore, blunted lipase and fat oxidase activities have been observed to be better than those of the microwave treatment [6].

Microwaving wheat buns for 20 s improve its texture, resulting in a softer, better quality buns [8]. Based on our previous research, microwave treatment for 20–30 s has been shown to improve the processing performance of whole wheat flour. Specifically, at 30 s, with a temperature of 57 °C, the treatment effectively reduced LA and LOX, thereby enhancing the flour’s quality and extending its shelf life. However, when the treatment duration was extended to 35 s, the temperature exceeded 68 °C, leading to damage to the gluten proteins and negatively affecting the flour’s processing characteristics. Therefore, this study considered the effects of synergistic microwave and steam treatment on the physicochemical properties of modified bran and whole wheat flour.

## 2. Results and Discussion

### 2.1. Component Analysis of Reconstituted Whole Wheat Flour

Composition is one of the important indicators for evaluating the quality of whole wheat flour. As shown in Table 1, the ash, dietary fiber, fat, and protein contents of the reconstituted whole wheat flour were significantly higher (*p* < 0.05) than those of the wheat flour group. S-WWF and MS-WWF had higher moisture contents owing to the introduction of external moisture. S-WWF exhibited the highest moisture content (12.3%). The steam treatment likely caused water molecules to move more vigorously due to the high temperature, which promoted the migration of moisture into the flour. Additionally, because the flour contained a high level of dietary fiber, it enhanced the water retention capacity of the starch, resulting in an increase in moisture content [9]. The ash content of the modified reconstituted whole wheat flour increased with increasing cooking time, with the highest ash content (1.94%) in MS-WWF4, which could be attributed to a reduction in moisture content. The decrease in fat content in reconstituted whole wheat flour may be attributed to fat oxidation under thermal conditions. In contrast, compared to U-WWF, the decrease in lipid content was more significant (*p* < 0.05) in S-WWF than in M-WWF, which may be due to the prolonged oxidation of lipids in air [10]. The reconstituted whole wheat flour had a dietary fiber content that ranged from 16.13 to 11.63%. In comparison, the reconstituted wheat flour had a significantly (*p* < 0.05) lower dietary fiber content (0.037%). This difference can be attributed to the addition of bran in all whole wheat flours at a 30% ratio, as bran contains a substantial amount of dietary fiber. Studies have shown that the dietary fiber content is more important due to its physiological functions, such as promoting gastrointestinal motility, reducing the absorption of cholesterol in the body, and facilitating the production of a feeling of satiety [11].

### 2.2. Color Parameters of Reconstituted Whole Wheat Flour

The color of whole wheat flour not only affects the appearance of the food but also has an impact on flavor and nutrition. Table 1 shows that both the microwave and steam treatment had a significant effect (*p* < 0.05) on the color parameters of the reconstituted powder. The L* values indicate the lightness and darkness of the reconstituted powder. The S-WWF treatment had a stronger effect on color than M-WWF. The heat treatment likely caused fat oxidation, which led to the Maillard reaction, resulting in increased darkness in the reconstituted flour [12]. Additionally, the extension of steam treatment time led to the lowest L* value and the highest a*, b*, C*, and ΔE values in MS-WWF4. The L* value and brightness of the recombinant powder significantly decreased (*p* < 0.05), whereas the a*, b*, and C* values significantly increased (*p* < 0.05). The powder appeared reddish and yellowish, which could be attributed to the high temperature and polyphenol oxidase activity. The recombinant powder underwent a Maillard reaction, resulting in darkening [13,14]. ΔE is primarily used to evaluate color changes in foods. The ΔE values of all the reconstituted whole wheat flours were significantly higher than that of the wheat flour group. Microwave treatment decreased ΔE more significantly than steam treatment. Furthermore, the ΔE increased significantly (*p* < 0.05) with longer time in the microwave combined with steam modification and was highest in MS-WWF4. This increase in ΔE can be attributed to a more pronounced Maillard reaction [15].

### 2.3. Swelling Power and Solubility of the Reconstituted Whole Wheat Flour

The solubility of whole wheat flour at a specific temperature indicates the strength of the interaction between the particles of whole wheat flour and water molecules. Dilatancy refers to the water absorption characteristics of whole wheat flour during gelatinization and its ability to retain water after centrifugation under specific conditions [16]. When swelling capacity and solubility decrease, the dough loses elasticity and extensibility. This negatively affects the process performance of the dough. As shown in Table 1, the solubility and swelling power of the reconstituted whole wheat flour group was significantly (*p* < 0.05) lower than that of the wheat flour. Compared to U-WWF, the solubility of S-WWF, MS-WWF1, MS-WWF2, and MS-WWF4 increased, whereas the solubility of M-WWF decreased. The solubility of MS-WWF3, on the other hand, remained relatively unchanged. With the extension of the treatment time, the increased penetration of water molecules into the interior of the starch, which led to an increase in solubility, had been attributed to the modification treatment. It disrupted the structure of the starch granules, allowing water molecules to penetrate the particles more easily and bind with the free hydroxyl groups in the starch through hydrogen bonds, forming monosaccharides that were more hydrophilic than the starch itself [17]. Compared to U-WWF, the expansion forces of S-WWF, MS-WWF1, MS-WWF2, MS-WWF3, and MS-WWF4 increased significantly (*p* < 0.05), whereas the expansion force of M-WWF decreased. The decrease in M-WWF swelling may be attributed to the degradation of branched-chain starch and starch-pasting at higher temperatures. This hinders water diffusion into the starch matrix, which is attributed to the presence of dextrinized starch and denatured proteins. The presence of lipids and proteins may affect SP. In the presence of lipids, the internal network of starch granules is enhanced, potentially inhibiting swelling. Additionally, heat treatment resulted in decreased protein and fat content. This phenomenon could explain the increase in the SP of reconstituted whole wheat flour following modification treatment.

### 2.4. Thermal Characterization of Reconstituted Whole Wheat Flour

In this present study, DSC was used to determine the To, Tp, Tc, and Hp values of different types of wheat flours, including untreated reconstituted whole wheat flour (U-WWF) and reconstituted whole wheat flour with various modified treatments [18].

Table 2 shows that To, Tp, Tc, and Hp values for the modified reconstituted whole wheat flour were smaller than that of U-WWF. The treatments had a positive impact on the untreated reconstituted whole wheat flour, with steam treatment being more effective than microwave treatment, reducing aging and improving taste. The temperature ranges for To, Tp, and Tc in the whole wheat flour group were 57.75–59.53 °C, 62.45–64.39 °C, and 66.98–68.97 °C, respectively. The Tp value of the combined treatment (microwave with steam treatment) was lower than that of a single treatment. This was likely due to the large amount of dietary fiber in the reconstituted flour, which exhibited strong water absorption and retention capacity under the combined modification treatment. Its binding ability with water molecules was stronger than that of the single-treated and untreated wheat starch groups. This influenced the water activity of the system, reducing the free water content in the gelatinization system, slowing down the swelling of the wet starch, and inhibiting gelatinization. As a result, the stability of the system was enhanced [19]. Additionally, Tp decreased initially and then increased with increasing steam time, with the lowest value recorded for MS-WWF3 (63.67 °C). The HP value of MS-WWF2 was the lowest among all experimental groups at 1.69 (J/g). These findings suggest that as the temperature increases, the crystal structure in the starch sample is progressively destroyed, resulting in a decrease in remaining crystal content. Consequently, the energy required to destroy the crystal structure is reduced, leading to a lower enthalpy value [20].

### 2.5. Rheological Properties of Reconstituted Whole Wheat Flour

The silty characteristics can reflect the processing characteristics of dough and the quality of the finished product to a certain extent. The rheological properties of the various modified whole wheat flours are presented in Table 2. The C1 parameter in Mixolab represents the water absorption of the flour, whereas C3 and C3-C4 reflect the strength of starch pasting during dough heating. The C5-C4 parameter demonstrates the regenerative nature of starch during cooling, and the C4/C3 ratio signifies the cooking performance of dough [21].

Compared to WF and U-WWF, with 58.4% and 67.7% water absorption, respectively, a significant (*p* < 0.05) increase in water absorption was observed in all modified groups, ranging from 68.7 to 69.5%. The highest water absorption was observed in MS-WWF4 (69.5%). The primary factors that influence water absorption are proteins, which can create a gluten mesh structure capable of absorbing large amounts of water. Additionally, the hydrocolloids (proteins, cellulose, and polysaccharides) present in wheat bran in reconstituted whole wheat flour contain numerous hydroxyl groups that bind more water molecules via hydrogen bond formation. This results in increased water absorption [22]. The stabilization time of the reconstituted whole wheat flour group was 6.10 min longer than that of the wheat flour group. This can be attributed to the addition of wheat bran, which resulted in a more sinewy and firmly bound dough that was less likely to open. Compared with U-WWF, M-WWF and S-WWF exhibited improved stabilization times. However, the effect of S-WWF was more significant, with the longest stabilization time recorded for MS-WWF2 (8.37 min). This could be attributed to the modification treatment, which positioned the disulfide bonds of glutenin in a manner that enhanced shear force resistance caused by stirring. Excessively long or short steaming times can weaken dough stability.

The C3 values of M-WWF and MS-WWF3 at 1.74 and 1.748 N/m, respectively, were lower than those of U-WWF. There was a negative correlation between C3 and broken starch content, indicating that a smaller C3 value allowed for a higher amount of broken starch for amylase utilization, resulting in a larger dough volume. The C3-C4 value was higher than that of U-WWF, with MS-WWF3 at 0.323 N/m. Additionally, there was a positive correlation between C3-C4 and amylase activity. This suggests that treatment can enhance the fermentation performance of dough [23]. The cooking stability of the dough, as indicated by the C4/C3 ratio, for both M-WWF and S-WWF was higher than that of U-WWF. Among them, M-WWF exhibited the highest stability value (0.864 N/m). MS-WWF was slightly less stable than M-WWF and S-WWF. Notably, MS-WWF4 exhibits a high stability value (0.837 N/m). The difference between C5 and C4 reflects the regenerative nature of starch during cooling. The results presented here are consistent with those presented in Section 2.4.

### 2.6. Microstructure of Reconstituted Whole Wheat Flour and Dough

**Figure 1:**
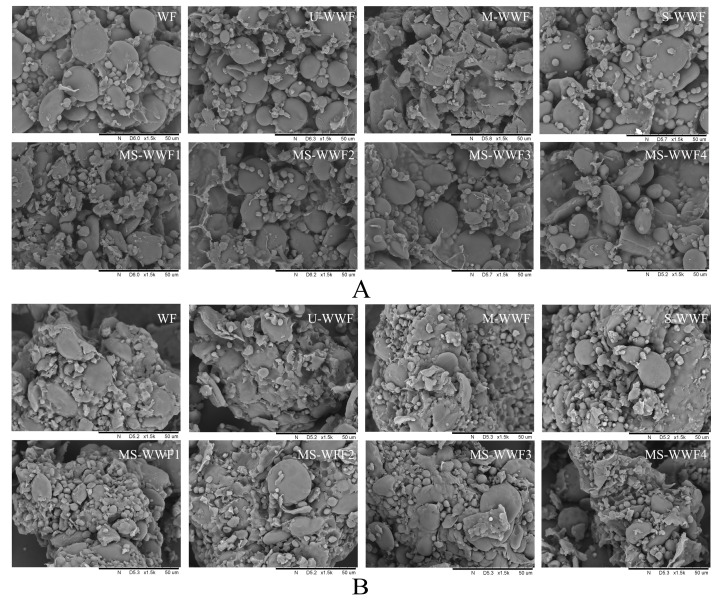
SEM images of reconstituted whole wheat flour and dough with different treatments: (**A**) SEM images of reconstituted whole wheat flour; (**B**) SEM images of reconstituted whole wheat dough. U-WWF, M-WWF, S-WWF, MS-WWF1, MS-WWF2, MS-WWF3, and MS-WWF4 represented four untreated, microwave, steam, and microwave followed by steam treatments for 5 min, 10 min, 15 min, and 20 min; WF represented wheat flour.

Electron microscopy images of wheat flour and reconstituted whole wheat flour subjected to various modified treatments are shown in Figure 1. The images revealed different starch granule shapes, including round and oval. From the perspective of overall structure, the treated wheat starches were subjected to varying degrees of damage. The gaps between starch granules were significantly reduced, aggregation occurred, and the interactions between starch granules were noticeably enhanced. In contrast to wheat flour, the reconstituted whole wheat flour groups showed starch granules covered by numerous bran granules. Starch had formed an interlocking and encapsulating structure with the bran, further enhancing the interaction between starch and dietary fiber. Since dietary fiber had a stronger hydrophilic ability than starch, it led to an increased swelling capacity of the starch [24]. This was attributed to the addition of bran granules to the reconstituted whole wheat flour. Furthermore, the granules of the modified reconstituted whole wheat flour, when compared to U-WWF, exhibited varying degrees of heat treatment-induced damage [25]. SEM imaging shows the network structure of all dough samples and reveals gluten-enveloping starch granules. However, the gluten network structure in the reconstituted whole wheat flour groups was not as uniformly wrapped as that in wheat flour with consistent air pores. This discrepancy can be attributed to the incorporation of a significant amount of bran particles into whole wheat flour, which disrupted the gluten network structure [26].

### 2.7. Crystalline Structure of Reconstituted Whole Wheat Flour and Dough

**Figure 2:**
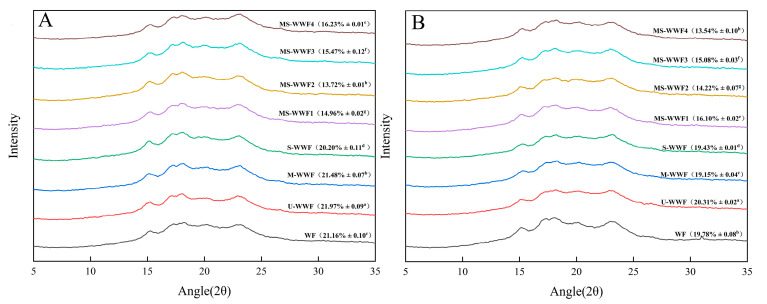
X-ray images of flour and dough of reconstituted whole wheat treated with different treatments: (**A**) X-ray images of reconstituted whole wheat flour; (**B**) X-ray images of reconstituted whole wheat dough. U-WWF, M-WWF, S-WWF, MS-WWF1, MS-WWF2, MS-WWF3, and MS-WWF4 represented four untreated, microwave, steam, and microwave followed by steam treatments for 5 min, 10 min, 15 min, and 20 min; WF represented wheat flour. Mean ± SD values in the same column with different superscript letters are significantly different (*p* < 0.05).

X-ray diffraction patterns revealed that the characteristics of microcrystals in a polycrystalline system are influenced by their size [27]. The X-ray diffraction pattern of the reconstructed whole wheat flour, illustrated in Figure 2, exhibited similarities, primarily featuring strong diffraction peaks at approximately 15°, 17°, 18°, and 23°. Notably, the double diffraction peaks were observed at 17° and 18°, whereas weaker diffraction peaks were observed near 20°. By analyzing the positions of these X-ray diffraction peaks, the crystalline nature of the sample can be classified as type A [28]. It is noteworthy that the intensity of the crystallization peak at 20° slightly increased in the modified treatment group, displaying a typical V-shaped diffraction peak. The appearance of the V-shaped diffraction peak were associated with the formation of amylose–lipid complexes [29]. During the processing, starch granules likely ruptured, causing the release of amylose. The high temperature promoted the complexation between amylose and lipids, leading to the formation of amylose–lipid complexes.

The grain starch in both groups consisted mainly of A-type crystals with a relatively compact structure. Upon heat treatment, the crystal properties of all the samples in the reconstituted whole wheat flour and dough groups remained A-type, indicating that the crystal shape did not change. Compared to the crystallinity of WF, the crystallinity of M-WWF and S-WWF decreased moderately after single modifications, while the crystallinity of MS-WWF1, MS-WWF2, MS-WWF3, and MS-WWF4 decreased significantly after combined modifications (microwave and steam). Detailed data are provided in Figure 2. This decrease in crystallinity may be attributed to the degradation of both the crystalline and non-crystalline regions within the starch particles following heat treatment [30].

### 2.8. Protein Secondary Structure of Reconstituted Whole Wheat Flour and Dough

**Figure 3:**
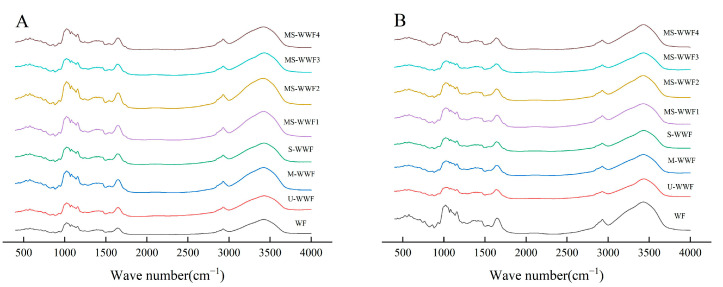
FTIR spectra of reconstituted whole wheat flour and dough under different treatments: (**A**) FTIR spectrum of reconstituted whole wheat flour; (**B**) FTIR spectrum of reconstituted whole wheat dough. U-WWF, M-WWF, S-WWF, MS-WWF1, MS-WWF2, MS-WWF3, and MS-WWF4 represented four untreated, microwave, steam, and microwave followed by steam treatments for 5 min, 10 min, 15 min, and 20 min; WF represented wheat flour.

Changes in secondary structure are essential for studying the structural properties of proteins. Proteins exhibit various absorption bands in their IR spectra, with the amide I band (1600–1700 cm^−1^) providing valuable information regarding protein secondary structures. These structures include α-helices, β-sheets, β-turn angles, and irregularly coiled structures [31]. These absorption bands, originating from the vibrations of the N–H and C = O bonds in the protein, reflected the characteristics of different secondary structures, as shown in Figure 3.

The FTIR profiles of reconstituted whole wheat flour and dough were in the range of 4000–400 cm^−1^. The protein secondary structure of reconstituted whole wheat flour is presented in Table 3. The formation of various secondary structures had been primarily stabilized by hydrogen bonds formed between the oxygen atoms in the carbonyl groups and the hydrogen atoms in the imino groups. Additionally, other forces, such as van der Waals forces and hydrophobic interactions, also contributed to the stabilization [32]. In the secondary structure, the α-helix served as the foundation of protein’s complex conformation and was also the most common regular secondary structure, being relatively ordered. The β-turns exhibited flexibility, allowing for movement within certain limits. In comparison to wheat flour, the reconstituted whole wheat flour exhibited a spiral form transformation of β-turn structure into an irregular curling and β-sheet structure, which is consistent with previous findings [33]. The addition of bran results in competitive water absorption, leading to the partial gluten dehydration. This redistribution of water in the dough affected the secondary structure of gluten proteins and disrupted their structural stability. Compared to U-WWF, the proportion of α-helix structures in M-WWF and dough reduced significantly. Furthermore, the α-helix content of S-WWF was lower than that of M-WWF, with the lowest α-helix content (12.27%) observed in MS-WWF3. Heat treatment restricted the aggregation of gluten polymers and enhanced the stretchability of gluten networks, which is consistent with previous findings [7]. Compared with U-WWF, the modified whole wheat flour exhibited a significantly (*p* < 0.05) higher β-sheet ratio, with S-WWF having a higher ratio than M-WWF. The reconstituted flour group had the highest β-sheet ratio (38.67% in MS-WWF4), whereas the dough group had a ratio of 45.09% in MS-WWF4. These results indicate that gluten possesses favorable viscoelasticity and baking quality.

### 2.9. Texture, Height–Diameter Ratio, and Specific Volume of the Steamed Bun

**Figure 4:**
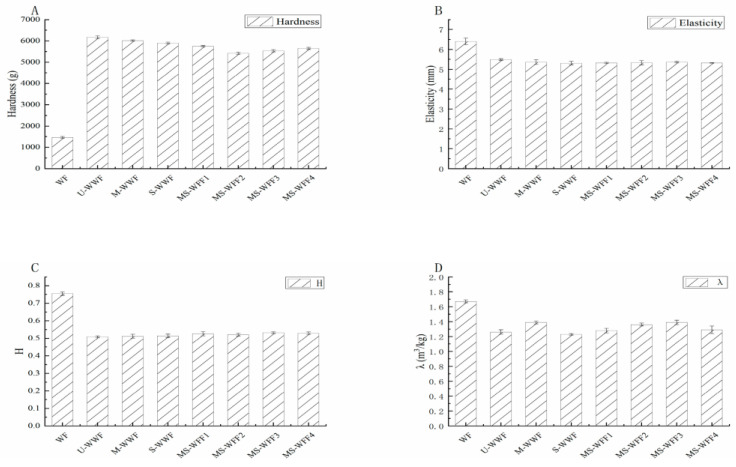
Eating quality images of flour and dough of reconstituted whole wheat treated with different treatments. (**A**) Hardness images of reconstituted whole wheat flour; (**B**) elasticity images of reconstituted whole wheat flour; (**C**) height-to-diameter ratio images of reconstituted whole wheat flour; (**D**) specific volume images of reconstituted whole wheat flour. U-WWF, M-WWF, S-WWF, MS-WWF1, MS-WWF2, MS-WWF3, andMS-WWF4 represented four untreated, microwave, steam, and microwave followed by steam treatments for 5 min, 10 min, 15 min, and 20 min; WF represented wheat flour. H: height-to-diameter ratio, λ: specific volume.

The hardness, elasticity, height-to-diameter ratio, and specific volume of the reconstituted whole wheat steamed buns subjected to different treatments are shown in Figure 4. Compared to U-WWF, the use of S-WWF resulted in a more significant reduction in bun hardness, whereas U-WWF had a relatively reduced impact. The steam treatment had likely increased the moisture content of the steamed buns compared to microwave treatment, resulting in better overall water retention, which led to a significant reduction in hardness. MS-WWF2 had the lowest hardness, with a reduction of 12.18% compared to U-WWF. Both steam and microwave treatments resulted in reduced steamed bun elasticity, with the steam treatment having a stronger effect on elasticity.

Additionally, both microwave and steam treatments increased the aspect ratio and specific volume of steamed buns. Among the treatments, MS-WWF3 exhibited the highest aspect ratio (0.532), whereas M-WWF had a higher specific volume than S-WWF. Furthermore, the specific volume of MS-WWF3 increased by 10.32% to 1.39 with U-WWF [34]. This is consistent with the experimental data from Mixolab, described in Section 2.5. Incorporating bran into flour reduced energy consumption, enhanced production efficiency, and improved sustainability. It also increased the nutritional value and functionality of whole wheat steamed buns, addressing the growing demand for health-conscious, nutritious, and convenient products. Furthermore, bran utilization optimized resource efficiency and reduced costs, demonstrating its significant potential for industrial-scale application.

## 3. Materials and Methods

### 3.1. Materials

Wheat flour was purchased from Yihai Kerry (Yihai Kerry Arawana Holdings Co., Ltd., Chuzhou, China) and wheat bran was purchased from Anhui Fengbao Grain and Oil Food Co., Ltd. (Fengyang, China).

### 3.2. Methods

#### 3.2.1. Wheat Bran Modification

In this experiment, 50 g of wheat bran with a moisture content of 12.40% ± 0.03 was contained in a glass disk, creating material that was approximately 1 cm thick. The wheat bran was subjected to microwave treatment in a microwave oven (P70D207L-D4; Galanz Microwave Household Appliance Manufacturing Co., Ltd., Zhongshan, China) at 700 W for 30 s. Subsequently, 100 g of microwave-treated wheat bran was placed on a steamer (material thickness of approximately 1 cm) and treated in a boiling water bath for 5, 10, 15, and 20 min (C21-RK2016, Midea Group Co., Ltd., Foshan, China). Finally, wheat bran was dried at room temperature.

The wheat bran was crushed using a cyclone mill (JXFM110; Jilin Dingli Steel Structure Co., Ltd., Jilin, China). Next, the crushed wheat bran was passed through an 80-mesh sieve and added to the wheat flour (at 30% proportion). The mixture was thoroughly mixed and stored at 4 °C. The final samples obtained were untreated whole wheat flour (U-WWF), whole wheat flour processed by microwave (700 w, 30 s) (M-WWF), whole wheat flour treated with steam for 5 min (S-WWF), and whole wheat flour processed by microwave (700 w, 30 s) followed by steam for 5 min (MS-WWF1), 10 min (MS-WWF2), 15 min (MS-WWF3), and 20 min (MS-WWF4).

#### 3.2.2. Preparation of Freeze-Dried Reconstituted Whole Wheat Dough

Thirty grams of reconstituted whole wheat dough was prepared from water obtained through Mixolab, as described in Section 3.2.7. The dough was initially frozen at −20 °C for 24 h and subsequently frozen at −80 °C for 48 h using a freeze dryer (LGJ-10FD, Beijing Songyuan Huaxing Technology Development Co., Ltd., Beijing, China). The samples were crushed, passed through a 100 mesh sieve, and stored in a dryer for determination, as described in Section 3.2.9 and Section 3.2.10.

#### 3.2.3. Gross Chemical Composition of Reconstituted Whole Wheat Flour

The moisture, ash, total protein, fat, and total dietary fiber content of the reconstituted whole wheat flour were determined using the Association of Official Analytical Chemists (AOAC) methods (AOAC 2000).

#### 3.2.4. Color Measurement of Reconstituted Whole Wheat Flour

The color parameters of the reconstituted whole wheat flour were measured using a CIE L*ab grade colorimeter (Hunterlab, Miniscan EZ, Reston, VA, USA).

#### 3.2.5. Swelling Power and Solubility of Reconstituted Whole Wheat Flour

The swelling power (SP) and solubility (S) of the samples were determined as previously described [35]^,^ with some modifications. Reconstituted whole wheat flour (1 g) was added to a 50 mL centrifuge tube along with 20 mL of deionized water. Next, the sample was shaken in a water bath at 150 r/min and 60 °C for 1 h. The centrifuge tube was removed from the water bath and the sample was allowed to cool to room temperature. Then, the samples were centrifuged at 1500× *g* for 15 min. The supernatant was discarded and the remaining sample was dried in an oven at 105 °C until a constant weight was achieved. The oven-dried samples were then weighed, and *S* and *SP* were calculated according to the following equations: Equations (1) and (2).

(1) $$S(\%)=\frac{A}{W}\times 100$$

(2) $$SP(\%)=P\times \frac{100}{W}\times (100-S)$$

where *A* is the mass of the supernatant after evaporation of a constant weight, *W* is the dry mass of the sample, and *P* is the mass of the precipitation after centrifugation.

#### 3.2.6. Heat Stability Characterization of Reconstituted Whole Wheat Flour

The thermodynamic properties of the samples were determined using a differential scanning calorimeter (DSC-3, Mettler Toledo Instrument (Shanghai) Co., Ltd., Zurich, Switzerland). Briefly, reconstituted whole wheat flour (3 mg) was mixed with distilled water (12 μL) in an aluminum crucible (40 μL), ensuring thorough mixing before sealing. The sample was then allowed to equilibrate at room temperature for 24 h. Using an empty pan as a reference, the sample was heated from 20 to 110 °C at a rate of 5 °C/min. The temperature at the beginning of pasting (To), the peak temperature (Tp), and the end temperature (Tc) were recorded, and the enthalpy (ΔH) was subsequently calculated.

#### 3.2.7. Rheological Characteristics of Reconstituted Whole Wheat Flour

The rheological properties of the reconstituted whole wheat flour were analyzed using a Mixolab analyzer (Mixolab 2, France Chopin Technology Co., Ltd., Paris, France). The Chopin+ standard protocol mode was used at a mixing speed of 80 rpm. The mix weight and water addition were adjusted to maintain the target torque C1 value at 1.10 ± 0.05 N·m. The default dough weight was 75 g, the wet base was 14%, and the water tank temperature was set at 30 °C.

#### 3.2.8. Determination of Microstructures of Reconstituted Whole Wheat Flour and Dough

The dry reconstituted whole wheat flour was weighed until a constant weight was achieved. The freeze-dried dough was cracked open, and samples of both constant-weight flour and small pieces of dough were affixed to double-sided tape on a short aluminum rod. Subsequently, a platinum film was applied under vacuum to both samples. The microstructures of the samples were observed using a scanning electron microscope (SEM) (TM3000, Hitachi Limited Co., Ltd., Tokyo, Japan) at an accelerating voltage of 20.0 kV.

#### 3.2.9. X-Ray Diffraction of Reconstituted Whole Wheat Flour and Dough

The reconstituted whole wheat flour was tightly packed in the drying balance with the freeze-dried dough sample in a rectangular silicon cell and is described in Section 3.2.2, as previously described [27]. X-ray diffraction analysis was performed using an X-ray diffractometer (X’Pert Pro, PANalytical, Almelo, The Netherlands) at 40 kV and 400 mA, with a scan range of 2 to 40° (2θ) and a scan rate of 3 °/min. The obtained X-ray patterns were analyzed using jade 6.5 software, and the relative crystallinity (*RS*) was calculated. The diffraction intensity of the entire sample included contributions from both crystalline and amorphous regions. The relative crystallinity (*RS*) was calculated by comparing the diffraction intensity of the crystalline portion with that of the entire sample. The relative crystallinity (*RS*) was determined using the following formula, as shown in Equation (3).

(3) $$RS(\%)=\frac{I_{C}}{I_{O}}\times 100$$

where *I_C_* is the diffraction intensity of the crystalline region in the sample (i.e., the X-ray diffraction peak intensity of the crystalline portion), and *I_O_* is the total diffraction intensity of the sample (i.e., the diffraction intensity, including both the crystalline and amorphous regions).

#### 3.2.10. Fourier Transform Infrared Spectroscopy (FTIR) of Whole Wheat Flour and Dough

The dried flour samples were mixed with the freeze-dried dough samples in a 1:100 (*w*/*w*) ratio with anhydrous KBr, as previously described [36]. The FTIR spectra were recorded within the range of 4000 cm^−1^ 400 cm^−1^, with a resolution of 4 cm^−1^, scan time of 16 s, and 32 scans using an FTIR spectrometer (Spectrum Two, PerkinElmer Co., Ltd., Massachusetts, MA, USA). The obtained FTIR spectra were analyzed using Peakfit v4 software, and the ratios of protein secondary structures were calculated based on the intensity of the absorption peaks. The relative proportions were calculated using the following formula, as shown in Equation (4).

(4) $$C(\%)=\frac{N}{M}\times 100$$

In the formula, *C* represents the secondary structure ratio of the protein (%), *N* denotes the integrated intensity of absorption peaks for the individual secondary structures (i.e., α-helix, β-sheet, random coil, and β-turn), and *M* represents the total integrated intensity of all secondary structure peaks.

#### 3.2.11. Edible Quality of the Steamed Bun

The reconstituted whole wheat flour (150 g) was mixed with 1.0% baking powder. Yeast (1.18%) was activated in warm water for 5 min. Subsequently, the appropriate amount of distilled water was added and the dough was kneaded and divided into four equal portions. The dough was fermented in a fermenter (LHP-160; Guangzhou Xuzhong Food Machinery Co., Ltd., Guangzhou, China) at 37 °C and 75% humidity for 40 min. Following fermentation, the dough was steamed for 30 min. The heat was turned off and the dough was allowed to simmer for 20 min with a closed lid. The dough was removed and dried for 10 min. It was used to calculate the height-to-diameter ratio (H) and specific volume (λ) of buns.

The steamed buns were sliced into 2 mm pieces, and their hardness and elasticity were measured using a TA4/1000 probe of the texture instrument (CT-3; Brookfield Co., Ltd., Middleborough, MA, USA). The test parameters included a trigger point value of −7 g, a test speed of 2 mm/s, and two cycles.

#### 3.2.12. Statistical Analysis

Statistical analyses were performed using the SPSS 21.0. One-way analysis of variance (ANOVA) and Duncan’s test were used to compare significant differences. Statistical significance was set at *p* < 0.05. Data were analyzed using Origin 8.0.

## 4. Conclusions

The modification of wheat bran by microwave irradiation combined with steam had a notable impact on the properties of reconstituted whole wheat flour and dough. With an increase in cooking time, the moisture and fat content decreased, whereas the ash, protein, and dietary fiber content increased. Additionally, there was a significant decrease in the L* value and brightness, as well as a significant increase in the a*, b*, and c* values and ΔE. These changes can be primarily attributed to the Maillard reaction. Solubility and swelling initially increased, followed by a decrease. This increase in solubility may be attributed to the disruption of the starch granule structure caused by the modification treatment. This disruption allows water molecules to penetrate the granules more easily and combine with the free hydroxyl groups in the starch via hydrogen bonding. Additionally, heat treatment reduced the fat content, leading to an increase in swelling power. The modified reconstituted whole wheat flour exhibited reduced To, Tp, Tc, and Hp values, indicating an improved anti-aging effect. The lowest Hp value (1.69 J/g) was achieved with a combined modification treatment (10 min microwave combined with steam modification).

Microwave combined with steam modification was found to enhance water absorption and stabilization time, particularly for C3, C3-C4, C4/C3, and C5-C4. This treatment also improved the fermentation performance and cooking stability of the dough. X-ray analysis revealed a significant decrease in starch crystallinity following the combined microwave and steam modification, which could be attributed to the degradation of both the crystalline and amorphous regions of the starch granules during heat treatment. Fourier spectroscopy analysis revealed that the spiral β-turn structure in the reconstituted whole wheat flour and dough underwent a transformation into an irregular curly–curly and β-sheet structure. The proportion of α-helix significantly decreased, while the proportion of β-sheet significantly increased in both the reconstituted whole wheat flour and dough. After 15 min of microwave-combined steam modification, the α-helix content reached its lowest point: 12.27% in the flour group and 14.43% in the dough group. After 20 min of microwave-combined steam modification, the β-sheet content reached its highest level: 38.67% in the flour group and 45.09% in the dough group. The hardness and elasticity of the buns had a significant impact on their overall quality, and MS-WWF2 had the best eating quality. In conclusion, modifying wheat bran with microwaves (700 W for 30 s) and steam (10 min) reduces processing costs and enhances the processing performance of reconstituted whole wheat flour. However, due to the high bran content (30%) in whole wheat flour, the shelf stability of the products is significantly compromised. Therefore, future research will focus on investigating the effects of thermal treatment on the storage quality of reconstituted whole wheat flour.

## Figures and Tables

**Table 1:** Composition content, color, S, and SPof reconstituted whole wheat flour with different treatments.

Samples	WF	U-WWF	M-WWF	S-WWF	MS-WWF1	MS-WWF2	MS-WWF3	MS-WWF4
Water (%)	12.4 ± 0.01 ^a^	12.1 ± 0.04 ^c^	11.7 ± 0.00 ^b^	12.3 ± 0.01 ^f^	12.1 ± 0.02 ^c^	12.0 ± 0.00 ^d^	12.0 ± 0.02 ^d^	11.9 ± 0.00 ^e^
Ash (%)	0.48% ± 0.00 ^e^	1.82% ± 0.00 ^d^	1.85% ± 0.00 ^c d^	1.86% ± 0.00 ^c^	1.88% ± 0.00 ^b c^	1.88% ± 0.00 ^b c^	1.91% ± 0.00 ^a b^	1.94% ± 0.00 ^a^
Fat (%)	5.82% ± 0.21 ^e^	9.55% ± 0.07 ^a^	9.54% ± 0.13 ^a^	9.01% ± 0.11 ^b^	9.06% ± 0.19 ^b^	8.38% ± 0.26 ^c^	8.15% ± 0.21 ^c d^	7.95% ± 0.16 ^d^
Protein (%)	8.93 ± 0.01 ^f^	11.26 ± 0.02 ^e^	11.70 ± 0.05 ^d^	12.04 ± 0.05 ^a^	11.73 ± 0.05 ^d^	11.91 ± 0.03 ^b^	12.02 ± 0.03 ^a^	11.85 ± 0.02 ^c^
Dietary fiber (%)	0.037 ± 0.13 ^g^	12.27 ± 0.15 ^e^	16.13 ± 0.20 ^a^	13.10 ± 0.10 ^d^	14.77 ± 0.21 ^b^	13.03 ± 0.15 ^d^	11.63 ± 0.15 ^f^	14.37 ± 0.19 ^c^
L*	95.49 ± 0.16 ^a^	87.58 ± 0.34 ^b^	87.19 ± 0.27 ^b c^	86.80 ± 0.11 ^d e^	86.65 ± 0.11 ^c d^	86.62 ± 0.17 ^d e^	86.22 ± 0.18 ^e^	86.23 ± 0.16 ^e^
a*	0.44 ± 0.10 ^d^	3.62 ± 0.13 ^c^	3.61 ± 0.05 ^c^	3.75 ± 0.14 ^c b^	3.74 ± 0.12 ^c b^	3.82 ± 0.04 ^b^	3.88 ± 0.11 ^b a^	4.04 ± 0.03 ^a^
b*	8.61 ± 0.16 ^g^	13.74 ± 0.07 ^e^	13.44 ± 0.15 ^f^	13.95 ± 0.09 ^c d^	14.27 ± 0.14 ^d^	14.46 ± 0.04 ^b c^	14.68 ± 0.19 ^b^	15.07 ± 0.06 ^a^
C*	8.63 ± 0.16 ^f^	14.21 ± 0.09 ^d e^	13.91 ± 0.14 ^e^	14.74 ± 0.21 ^c^	14.56 ± 0.17 ^c d^	14.96 ± 0.05 ^b c^	15.19 ± 0.21 ^b^	15.70 ± 0.24 ^a^
ΔE	9.69 ± 0.21 ^d^	18.70 ± 0.41 ^c^	18.73 ± 0.11 ^c^	19.70 ± 0.21 ^b^	19.47 ± 0.27 ^b^	19.88 ± 0.13 ^b^	20.32 ± 0.26 ^a^	20.71 ± 0.12 ^a^
S ^1^ (%)	15.80 ± 0.17 ^a^	14.65 ± 0.23 ^d^	13.86 ± 0.65 ^e^	15.06 ± 0.26 ^c^	15.03 ± 0.19 ^c^	15.20 ± 0.27 ^b^	14.66 ± 0.21 ^d^	15.16 ± 0.18 ^b^
SP ^2^ (%)	4.24 ± 0.01 ^a^	3.71 ± 0.03 ^e^	3.61 ± 0.12 ^f^	4.05 ± 0.03 ^d^	4.06 ± 0.04 ^d^	4.12 ± 0.02 ^c^	4.17 ± 0.12 ^b^	4.12 ± 0.03 ^c^

U-WWF, M-WWF, S-WWF, MS-WWF1, MS-WWF2, MS-WWF3, and MS-WWF4 represented four untreated, microwave, steam, and microwave followed by steam treatments for 5 min, 10 min, 15 min, and 20 min; WF represented wheat flour. Assays were performed in triplicate. Mean ± SD values in the same column with different superscript letters are significantly different (*p* < 0.05). ^1^ S: solubility. ^2^ SP: swelling power.

**Table 2:** Thermal properties and rheological properties of reconstituted whole wheat flour with different treatments.

Samples	WF	U-WWF	M-WWF	S-WWF	MS-WWF1	MS-WWF2	MS-WWF3	MS-WWF4
To (°C)	57.75 ± 0.03 ^h^	59.53 ± 0.65 ^a^	59.50 ± 0.06 ^b^	59.48 ± 0.01 ^c^	59.26 ± 0.00 ^d^	59.21 ± 0.03 ^e^	59.07 ± 0.04 ^g^	59.18 ± 0.00 ^f^
Tp (°C)	62.45 ± 0.01 ^h^	64.39 ± 0.05 ^a^	64.23 ± 0.07 ^c^	64.27 ± 0.00 ^b^	63.92 ± 0.01 ^e^	63.77 ± 0.00 ^f^	63.67 ± 0.05 ^g^	63.93 ± 0.04 ^d^
Tc (°C)	66.98 ± 0.02 ^g^	68.97 ± 0.05 ^a^	68.89 ± 0.14 ^c^	68.91 ± 0.00 ^b^	68.73 ± 0.03 ^d^	68.54 ± 0.02 ^e^	68.29 ± 0.03 ^f^	68.54 ± 0.05 ^e^
Hp (J/g)	3.45 ± 0.01 ^a^	2.60 ± 0.04 ^c^	1.84 ± 0.00 ^g^	2.59 ± 0.13 ^d^	2.32 ± 0.02 ^f^	1.69 ± 0.10 ^h^	2.34 ± 0.02 ^e^	2.89 ± 0.05 ^b^
WA (%) ^1^	58.4 ± 0.00 ^g^	67.7 ± 0.00 ^f^	68.7 ± 0.00 ^e^	68.9 ± 0.00 ^c^	68.8 ± 0.00 ^d^	69.4 ± 0.00 ^b^	69.4 ± 0.00 ^b^	69.5 ± 0.00 ^a^
ST (min) ^2^	6.100 ± 0.01 ^d^	7.400 ± 0.20 ^c d^	7.533 ± 0.12 ^a^	7.767 ± 0.61 ^e^	7.500 ± 0.36 ^e^	8.367 ± 0.35 ^a b^	7.733 ± 0.06 ^b c d^	7.900 ± 0.10 ^b c^
C1 (N/m)	1.119 ± 0.01 ^a^	1.118 ± 0.01 ^a^	1.089 ± 0.00 ^b c^	1.115 ± 0.03 ^a b^	1.108 ± 0.00 ^a b^	1.068 ± 0.00 ^c^	1.086 ± 0.01 ^b c^	1.118 ± 0.01 ^b^
C3 (N/m)	1.804 ± 0.00 ^a^	1.771 ± 0.01 ^b^	1.740 ± 0.01 ^c^	1.797 ± 0.01 ^a^	1.762 ± 0.02 ^b^	1.756 ± 0.01 ^b c^	1.748 ± 0.02 ^b c^	1.763 ± 0.02 ^b c^
C5-C4 (N/m)	1.061 ± 0.01 ^a^	0.834 ± 0.16 ^b c^	0.650 ± 0.02 ^c^	0.821 ± 0.15 ^b c^	0.735 ± 0.14 ^b c^	0.694 ± 0.01 ^c^	0.769 ± 0.18 ^b c^	0.946 ± 0.01 ^b^
C3-C4 (N/m)	0.060 ± 0.00 ^h^	0.317 ± 0.01 ^b^	0.237 ± 0.01 ^g^	0.290 ± 0.01 ^e^	0.299 ± 0.02 ^d^	0.307 ± 0.01 ^c^	0.323 ± 0.01 ^a^	0.288 ± 0.02 ^f^
C4/C3	0.967 ± 0.01 ^a^	0.834 ± 0.00 ^c^	0.864 ± 0.01 ^b^	0.839 ± 0.01 ^c^	0.830 ± 0.01 ^c d^	0.825 ± 0.01 ^c d^	0.819 ± 0.02 ^d^	0.837 ± 0.01 ^c^

U-WWF, M-WWF, S-WWF, MS-WWF1, MS-WWF2, MS-WWF3, and MS-WWF4 represented four untreated, microwave, steam, and microwave followed by steam treatments for 5 min, 10 min, 15 min, and 20 min; WF represented wheat flour. Assays were performed in triplicate. Mean ± SD values in the same column with different superscript letters are significantly different (*p* < 0.05). ^1^ WA: water absorption. ^2^ ST: stability time.

**Table 3:** Protein secondary structure of reconstituted whole wheat flour and dough.

Samples		β-Sheet (%)	Random Coil (%)	α-Helix (%)	β-Turn (%)
The reconstituted whole wheat flour	WF	30.66 ± 0.03 ^h^	16.68 ± 0.01 ^f^	16.10 ± 0.02 ^e^	36.56 ± 0.02 ^a^
	U-WWF	32.37 ± 0.01 ^e^	18.01 ± 0.02 ^b^	17.56 ± 0.04 ^a^	32.06 ± 0.08 ^e^
	M-WWF	37.81 ± 0.02 ^c^	14.59 ± 0.00 ^h^	14.87 ± 0.01 ^f^	32.73 ± 0.03 ^d^
	S-WWF	39.82 ± 0.09 ^a^	16.76 ± 0.01 ^e^	13.23 ± 0.03 ^g^	30.18 ± 0.04 ^g^
	MS-WWF1	31.19 ± 0.00 ^g^	17.71 ± 0.06 ^d^	17.15 ± 0.06 ^c^	33.94 ± 0.00 ^b^
	MS-WWF2	31.59 ± 0.03 ^f^	17.82 ± 0.07 ^c^	17.47 ± 0.01 ^b^	33.12 ± 0.01 ^c^
	MS-WWF3	34.82 ± 0.06 ^d^	22.25 ± 0.01 ^a^	12.27 ± 0.02 ^h^	30.66 ± 0.02 ^f^
	MS-WWF4	38.67 ± 0.02 ^b^	16.20 ± 0.03 ^g^	16.32 ± 0.01 ^d^	28.81 ± 0.01 ^h^
The reconstituted whole wheat dough	WF	34.19 ± 0.06 ^f^	18.53 ± 0.03 ^a^	14.04 ± 0.04 ^g^	33.24 ± 0.02 ^a^
	U-WWF	36.35 ± 0.02 ^e^	14.15 ± 0.02 ^g^	16.81 ± 0.03 ^c^	32.69 ± 0.01 ^d^
	M-WWF	37.81 ± 0.01 ^d^	14.59 ± 0.09 ^e^	14.87 ± 0.08 ^d^	32.73 ± 0.07 ^c^
	S-WWF	39.66 ± 0.04 ^b^	14.66 ± 0.04 ^d^	14.01 ± 0.01 ^h^	31.67 ± 0.03 ^e^
	MS-WWF1	32.25 ± 0.01 ^h^	17.86 ± 0.02 ^c^	17.13 ± 0.05 ^a^	32.76 ± 0.02 ^b^
	MS-WWF2	33.61 ± 0.03 ^g^	17.99 ± 0.03 ^b^	17.04 ± 0.01 ^b^	31.36 ± 0.05 ^f^
	MS-WWF3	38.46 ± 0.02 ^c^	13.86 ± 0.04 ^h^	14.43 ± 0.05 ^f^	33.24 ± 0.01 ^a^
	MS-WWF4	45.09 ± 0.03 ^a^	14.55 ± 0.01 ^f^	14.70 ± 0.02 ^e^	25.66 ± 0.00 ^g^

U-WWF, M-WWF, S-WWF, MS-WWF1, MS-WWF2, MS-WWF3, and MS-WWF4 represented four untreated, microwave, steam, and microwave treatments followed by steam for 5 min, 10 min, 15 min, and 20 min; WF represented wheat flour. Assays were performed in triplicate. Mean ± SD values in the same column with different superscript letters are significantly different (*p* < 0.05).

## Data Availability

We promise that the data in this paper come from our experiments, which are reliable. The dataset analyzed during this current study are available from the corresponding author on reasonable request.
